# Multidimensional Information Encryption and Storage: When the Input Is Light

**DOI:** 10.34133/2021/7897849

**Published:** 2021-01-09

**Authors:** Senyang Liu, Xiaohu Liu, Jinying Yuan, Jie Bao

**Affiliations:** ^1^Department of Electronic Engineering, Tsinghua University, Beijing 100084, China; ^2^Key Laboratory of Organic Optoelectronics & Molecular Engineering, Department of Chemistry, Tsinghua University, Beijing 100084, China

## Abstract

The issue of information security is closely related to every aspect of daily life. For pursuing a higher level of security, much effort has been continuously invested in the development of information security technologies based on encryption and storage. Current approaches using single-dimension information can be easily cracked and imitated due to the lack of sufficient security. Multidimensional information encryption and storage are an effective way to increase the security level and can protect it from counterfeiting and illegal decryption. Since light has rich dimensions (wavelength, duration, phase, polarization, depth, and power) and synergy between different dimensions, light as the input is one of the promising candidates for improving the level of information security. In this review, based on six different dimensional features of the input light, we mainly summarize the implementation methods of multidimensional information encryption and storage including material preparation and response mechanisms. In addition, the challenges and future prospects of these information security systems are discussed.

## 1. Introduction

Information security has become a significant global issue not only in economic and military fields but also in the daily lives. Because of the large temptation of enormous profits which can be potentially obtained from the acquisition of secured information, the methods for counterfeiting or illegal decryption emerge endlessly [1–3]. There is thus a fierce arms race between protecting and counterfeiting information, which on the other hand promotes the rapid development of information security technology to some extent [4]. Information encryption is an effective way to provide the security of information, and the encrypted information is stored in the materials [5, 6]. Only with the correct “key”, the reliable information can be decrypted. In ancient China, when half of “Hu-Fu” (a tiger-shaped tally issued to generals for troop movement) delivered by the messenger could match perfectly with the other half belonging to the general, the general could judge that it was the authentic military order from the emperor. The information representing “trust” was encrypted and stored in the “Hu-Fu.” Analogously, in modern society, anticounterfeiting labels are widely used to ensure information security [7–9]. Hence, information security technologies with both encryption and storage abilities are always in demand, which attracts tremendous interests for researchers.

A system of information encryption and storage can be simplified as the integration of the input, the material, and the output [10]. The system of single-dimension information encryption and storage, with one input, one material, and one output, merely exhibits a single transition between two states of “0” and “1.” The relatively less secured information is thus easier to be cracked by brute-force attacking which requires little effort to find the correct solution illegally. Furthermore, information encrypted and stored in this way may be duplicated or modified, resulting in confusion in justifying the authenticity of information. To increase the security of information, one of the methods is to design an optical authentication system based on physical unclonable functions [11–13]. It can be generally considered a system composed of materials with unique properties that each material corresponds to a single input and a single output. The intrinsic, unique, and random characteristics of materials originate from the nondeterministic process during the preparation, which makes it impossible to be duplicated by definition. Another way is to achieve multidimensional information encryption and storage in one (composite) material. This method can not only dramatically increase the difficulty of counterfeiting but also expand the capacity of information storage [14–16]. From the view of the above-mentioned system consisting of the input, the material, and the output, the multiple dimensions of information can correspond to the multidimensional input or the multidimensional output to provide a high level of information security. In the output side, multidimensional information usually displays in the form of light, which can be further classified into the dimensions of wavelength [17, 18], spectra [19, 20], time [21, 22], viewing angle [23, 24], or spatial position [25, 26]. More interesting works are based on the diversity at the input side that can be in the form of light [27–31], thermal [32, 33], electricity [34], or chemical reagents [35–37]. The diversity of the input can (1) provide more choices, which is a great advantage for multidimensional information encryption and storage, and (2) influence the two trailing parts—the material and the output—in multiple ways.

Light, as the most common physical quantity in daily life, is one of the promising candidates as the input owing to its easy accessibility and high-level controllability [38, 39]. The features of light, such as wavelength, duration, phase, polarization, depth, and power, can all be regarded as different dimensions of the input. Based on the original system of single-dimension information, multidimensional information encryption and storage can be achieved by adding one or more of the features of light [31, 40]. By choosing a light source and designing a light path properly, the above-mentioned six features of light can be well controlled to obtain the desired input light. Then, it will be more purposeful for the preparation and property optimization of the materials. Previous reviews related to information encryption and storage using the light as the input are mostly summarized in the view of materials such as upconversion materials [41–43], structural color materials [44, 45], and metasurface materials [40, 46]. Although the synthesis, property, and application of these materials are introduced in detail, the intrinsic connection of different materials has not been fully illustrated, especially for the role of light in the field of multidimensional information encryption and storage.

**Figure 1:**
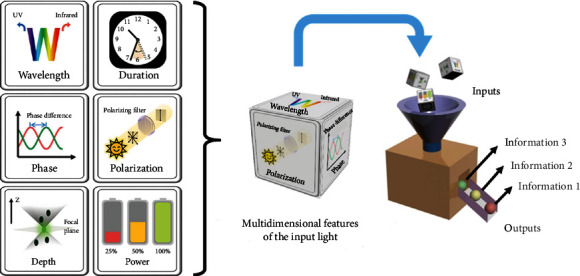
Multidimensional features of light as the input applied for multidimensional information encryption and storage.

In this review, we will focus on light itself as the sole input, to summarize how it plays the role in the multidimensional information encryption and storage. As shown in Figure 1, there are six dimensional features of light to produce different inputs, including wavelength, duration, phase, polarization, depth, and power. Each cube dice representing the specific input signal is thrown into a magic box where multidimensional information has been encrypted and stored. The distinct color globules to roll out are considered different information outputs. The complete information can be read out when the box matches well with the added dices. In the following sections, we will start on the selection of the dice and then design the corresponding box for information encryption and storage based on the features of the dices. The advantages and disadvantages of every type of dice are estimated at the end of each section. Finally, a summary and perspectives about multidimensional information encryption and storage when the input is light are proposed for future development. It should be noteworthy that light is considered the sole input in this review. The combination of light and other inputs is excluded due to the limited space. Related contents can be found elsewhere [47–53].

## 2. Light: Wavelength

Conceptually, light includes not only visible light within the range of 400 nm to 700 nm that can be perceived by human eyes but also nonvisible light like ultraviolet (UV) light and near-infrared (NIR) light. Light of different wavelengths as the input means discrepant energy inputs. Based on the rule of energy matching, they could induce different physical or chemical changes of materials, which further exhibit the encrypted and stored multidimensional information. For information encryption and storage, whatever the input is, the most convenient output is in the form of visible light that could be easily read out by the naked eye, smartphone, or miniature spectrometer [54]. Hence, when the input is light of different wavelengths, the key problem is mainly to prepare optical materials to achieve the wavelength transformation for outputting the desired visible light. For the case of transmission or reflection of light, with the same wavelength for the input light and the output light, we will discuss in later sections. According to the difference of wavelength transformation, the optical process can be mainly divided into two classes: downconversion luminescence (DCL) and upconversion luminescence (UCL).

### 2.1. DCL

**Figure 2:**
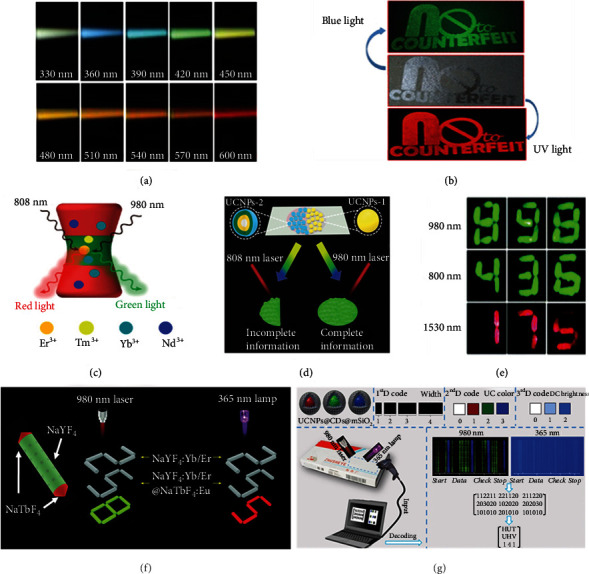
(a) Emission photographs of the excitation-dependent CDs excited from 330 nm to 600 nm. (b) Dual-luminescent pattern of the security ink composed of the coordination compound of europium(III) and fluorescein under blue light and UV light irradiation. (c) Orthogonal emissive properties of the core-shell-shell structured UCNPs. (d) Schematic illustration of inks composed of two kinds of UCNPs showing incomplete and complete information at 808 nm and 980 nm, respectively. (e) Different numbers observed under the excitation wavelength of 980 nm, 800 nm, and 1530 nm. (f) The structure of tip-modified NaYF_4_@NaTbF_4_ microrods and their dual-mode information displayed under a 980 nm laser and a 365 nm lamp. (g) The security application of 3D anticounterfeiting barcodes composed of sandwiched nanohybrids. Reproduced with permissions: (a) from [58], Copyright 2015, Wiley-VCH; (b) from [59], Copyright 2018, American Chemical Society; (c) from [63], Copyright 2019, American Chemical Society; (d) from [60], Copyright 2017, The Royal Society of Chemistry; (e) from [27], Copyright 2017, The Royal Society of Chemistry; (f) from [65]. Copyright 2018, The Royal Society of Chemistry; (g) from [66], Copyright 2019, American Chemical Society.

DCL means the optical process that produces longer-wavelength emission when exposed under shorter-wavelength excitation, with a change from the high-energy photon to the low-energy one. In general, DCL materials are excited by UV light or short-wavelength visible light like violet or blue light. Most luminescent materials including organic luminescent dyes, aggregation-induced emission (AIE) molecules, and semiconductor quantum dots are all regarded as the typical DCL materials [55–57]. Based on the distinguishable changes in emission spectra under different excitation wavelengths, different information can be encoded corresponding to each input light. Compared with the change of emission intensity, the change in peak position attracts more interests as the color change is more suitable for multidimensional information encryption and storage. Given the fact of multiple emission centers of carbon dots (CDs), CDs are the typical materials to display excitation-dependent properties. When utilizing light with different excitation wavelengths from 330 nm to 600 nm as the input, multiple DCL colors across the entire visible spectrum could be seen (Figure 2(a)) [58]. Besides, owing to comparable DCL intensities for different inputs, it was achievable to keep each piece of information clearly observable.

Alternatively, another simpler approach for multidimensional information encryption and storage is to assemble multiple DCL materials with different excitation and emission properties. A coordination compound of europium(III) and fluorescein was selected to form a dual luminescent ink for printing on the substrates [59]. Under UV light of 367 nm, the former played a dominant role to emit red fluorescence and the emission intensity of the latter could be neglected (Figure 2(b)), while under blue light of 445 nm, roles were reversed that the green fluorescent pattern produced by fluorescein was observed. Thus, according to the mechanism of DCL, dual-state information was encrypted and stored through two input light of different wavelengths.

### 2.2. UCL

UCL refers to the optical process that shorter-wavelength emission appears when excited by longer-wavelength light (usually in the form of NIR-to-*vis* conversion). For UCL materials, different Ln^3+^ ions doped into one host material can play the role as sensitizers to harvest photons from the input light or activators to emit different-wavelength light, respectively. Owing to the specificity of UCL from the low-energy photon to the high-energy one, it shows the great advantage of avoiding background fluorescent interference. Thus, there are more choices of substrates to provide the high-quality information output under different conditions [60].

By adjusting the host lattices and dopant ions during the chemical synthesis, upconversion nanoparticles (UCNPs) with two desired excitation or emission wavelengths can be easily prepared [61, 62]. For the purpose of emitting two distinct photoluminescence under 980 nm and 808 nm, respectively, the core-shell-shell structured UCNPs with multiple ion doping (NaErF_4_:Yb/Tm@NaYF_4_:Yb@NaNdF_4_:Yb) were synthesized. Based on the specific energy migration pathways of UCNPs, the whole Yb^3+^ sensitizers played the role of absorbing the excitation energy when excited at 980 nm. The excited-state electron could then transfer to Er^3+^ in the core, leading to red emission at 650 nm. Whereas at 808 nm excitation wavelength, only Nd^3+^ in the outer shell could harvest the excitation energy at first. Immediately, Er^3+^ in the core accepted the energy that transferred from Yb^3+^, emitting green light at 540 nm (Figure 2(c)) [63]. The dual-excitation UCNPs could be assembled with the usual UCNPs with single emission peak for patterning. As shown in Figure 2(d), under 980 nm, both UCNPs could be excited to display complete information, whereas only one of them could work under 808 nm excitation with incomplete information [60].

Through the precision synthesis of UCNPs to obtain the desired UCL performance under every excitation wavelength, the amount of information encrypted and stored can further increase [64]. One of the limitations to influence effective UCL is the concentration quenching effect of an activator. The harmful cascade energy migration and cross-relaxation between dopant ions result from high-concentration doping which raises the possibility of excited-state energy trapped by the quenching sites. In order to realize a “quenching site-free” system, the core-shell nanostructured UCNPs (NaErF_4_@NaYF_4_) were fabricated, where the optimal concentration of Er^3+^ could increase to 100 mol% [27]. The UCL material showed three-band excitation and monochromic red emission. After introducing the other two materials with a low-doping activator that only performed effectively on one specific excitation wavelength, three numbers were printed with three inks layer by layer. Under different excitation wavelengths within the range of NIR (980 nm, 800 nm, or 1530 nm), each piece of information appeared corresponding to the specific ink (Figure 2(e)). Therefore, better understanding of the UCL mechanisms for rational design of UCNP structures is of great help to encrypting and storing multidimensional information in multichannel excitation.

### 2.3. Combination of DCL and UCL

Apart from the monomode luminescent process of DCL or UCL, the dual-mode luminescence by combining both is also used for multidimensional information encryption and storage. From the practical point of view, the dual-mode luminescent process can be realized in a single material [67, 68] or separated materials [69], while the former is more difficult to imitate illegally. It is essential to design structure precisely for arranging UCL and DCL modules in different spatial regions.

For the rod-like structures with a dual mode, a UCL bar with two DCL tips was designed and prepared by multistep growth (Figure 2(f)) [65]. Different optical processes were precisely confined in separated areas. After printing with another monomode luminescent material, overlapped green “8” displayed under a 980 nm laser, while only dual-mode microrods showed luminescence under a 365 nm lamp (red “5”). With regard to nanoparticles, it is a good way to assemble DCL and UCL materials by designing core-shell structures. For instance, CDs and UCNPs could be encapsulated into mesoporous silica to form a sandwich structure (UCNPs@CDs@mSiO_2_) (Figure 2(g)) [66]. The luminescence property of an end product was dependent on individual luminescent material. Thus, three kinds of nanohybrids were successfully synthesized, with blue emission under UV excitation and three emissions (red, green, and blue) under NIR excitation. On the basis of the original 1D space distribution encryption, information concealed under 980 nm and 365 nm was considered the 2^nd^- and 3^rd^-dimensional encryption, which finally generated 3D luminescent barcodes. The differences of UCL colors and DCL brightness were marked as particular numbers. Under 365 nm UV light and 980 nm laser, 3D digit information could be further encoded for more complex information.

Besides the DCL or UCL of luminescent material, nonlinear wavelength conversions of light could also be used for multidimensional information encryption and storage. The authentic image could be read out only when the wavelength of the input light met the requirement to produce the second harmonic generation signal [70]. Theoretically, by utilizing more types of materials or multiluminescent materials under different excitation wavelengths, it could further increase the amount of available information and improve the security level. Nevertheless, owing to the wavelength diversity of the input light, it is unavoidable to use more intricate light sources to meet the requirements of different input light. It may become a limitation for its future practical uses without the development of integrated light sources simultaneously. Another problem is that the information printed by multiluminescent materials may be counterfeited by the uniform mixing of several single-luminescent materials [71]. The superficial resemblances make it difficult to judge the authenticity of information. Therefore, the wavelength tends to combine with other dimensions of light to provide a high-level information security.

## 3. Light: Duration

Duration, regarded as irradiation time, is further understood as controllable changes of luminescent property under the irradiation for various periods of time. Multidimensional information is therefore encrypted and stored in the dimension of time, which is highly related to photoresponsive materials. Under light irradiation, the spectra of photoresponsive materials are dynamically changing, showing the shifting of absorption/emission peak or the increase (decrease) in intensity [72, 73]. The differences of spectra originate from the structure transformation of photoresponsive materials under different irradiation durations, which can be encoded as multidimensional information for encryption and storage. According to the different mechanisms of photoresponsive reaction, it can be mainly divided into two parts: irreversible reactions and reversible reactions.

### 3.1. Irreversible Reactions

Irreversible reactions related to light are usually based on either the photoinduced decomposition by cleaving functional groups or the photoinduced addition/polymerization to form stable covalent bonds. Different irradiation durations indicate the differences in the extent of reaction.

**Figure 3:**
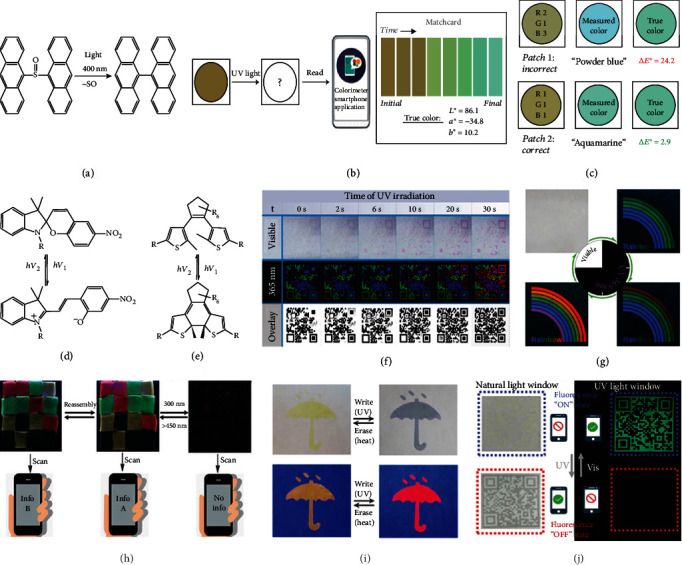
(a) The photoinduced decomposition reaction of AnSO under 400 nm. (b) The process for product authentication by a smartphone app based on the color changes with time. (c) Comparison of testing results between two patches with one incorrect and another correct. Reversible reactions based on photoinduced isomerization of (d) SP and (e) DAE. (f) Dynamic changes with different UV irradiation durations of a QR code image composed of cellulose-based trichromatic materials under 365 nm and visible light. (g) The reversible color change loop of the fluorescent rainbow over time. (h) Information readout of the reassembled and encrypted fluorescent pattern with four kinds of hydrogels under 254 nm UV light. (i) Four umbrella-shape fluorescence patterns composed of DSA-2SP as the anticounterfeiting label displayed under the corresponding input. (j) The dual decryption process of the dynamic fluorescent anticounterfeiting QR code printed with liquid crystal nanoparticle-based ink. Reproduced with permissions: (a) from [74], Copyright 2016, Wiley-VCH; (d, e) from [73], Copyright 2012, The Royal Society of Chemistry; (f, g) from [28], Copyright 2017, Wiley-VCH; (i) from [79], Copyright 2017, American Chemical Society; (j) from [80], Copyright 2019, Wiley-VCH.

Taking the photoinduced decomposition as an example, it is of great importance to control the rate of decomposition precisely in order to keep just a piece of information for an irradiation time. The controllable photoresponsive transition of 9,9′-bis(anthracene) sulfoxide (AnSO) made it a promising candidate for multidimensional information encryption and storage [74]. After extruding the SO bridge between anthracene units under UV irradiation, the luminescent molecules with blue light, 9,9′-bianthryl, was yielded quantitatively (Figure 3(a)). On the basis of AnSO structure, red and green fluorescence molecules could also be achieved by modifying triarylamine and phenothiazine at 10,10′-positions of the original dianthracene hosts, respectively [75]. To illustrate the three-color photoresponsive materials applied in the multidimensional information encryption and storage, a controlled experiment between products with the correct RGB ratio (1 : 1 : 1) and with the incorrect one (2 : 1 : 3) was carried out. During UV light irradiation, the color captured and analyzed by a smartphone application was dynamically changing (Figure 3(b)). Although both initial fluorescent colors were the same (orange-brown), the final colors were completely distinct (powder blue and aquamarine) (Figure 3(c)), which could be utilized to determine whether the product was authentic or not. Apart from the contrast in the final fluorescent color, it is possible to make full use of the entire process of dynamic fluorescent change to encrypt into different information. Owing to the irreversibility of photoinduced decomposition, the complete information along the time dimension is destroyed after the first reading, which keeps the information highly secured and difficult to counterfeit.

### 3.2. Reversible Reactions

For the reversible reactions, photoinduced isomerization is the most remarkable characteristic, in which organic molecules reversibly change the structure upon irradiation in the form of the *cis*-*trans* isomerism or the tautomerism [76]. Among them, spiropyran (SP) and diarylethene (DAE) are two major categories of compounds for the multidimensional information encryption and storage.

The photochromic processes of SP and its derivatives present a controllable change in the spectra, which has been widely studied in optical information storage [77, 78]. In general, they are colorless in the form of the closed spiro, while it can transform to the open spiro to emit light after UV light irradiation (Figure 3(d)) [73]. To illustrate multidimensional information encrypted and stored in the dimension of time, SP, fluorescein, and pyrene were selected as fluorescent groups to couple with cellulose skeletons to prepare materials with tunable full-color emission [28]. After mixing the trichromic materials to establish an effective fluorescence resonance energy transfer (FRET) system, a photochromism phenomenon with reversibility and nonlinearity was observed with irradiation duration under 365 nm. Because of the photoinduced isomerization of SP, the absorption (or excitation) and emission spectra of SP-based materials changed dynamically. It resulted in the change of FRET efficiency (spectroscopic overlap) between green/blue and red color components. Therefore, along with the irradiation time, the ratio of red emission increased, while the ratios of green and blue decreased on different levels. An entire quick response (QR) code image could be seen after 30 s UV irradiation to display the correct information (Figure 3(f)). Owing to the reversibility of photoinduced isomerization, the information of color change loop such as the rainbow pattern of different colors could be read out repeatedly by choosing correct irradiating duration (Figure 3(g)).

In contrast to SP, the role of DAE is to quench fluorescence with irradiation duration, due to the structure change from the open form to the closed one by the tautomerism [73, 81, 82] (Figure 3(e)). Hence, it is essential to introduce other fluorescent material as a luminescent center. Ln^3+^ coordination compounds were then selected, together with DAE, to be added into the polymerization reaction for the formation of photocontrollable fluorescent hydrogels [83]. By tuning Ln^3+^ and the DAE unit, four types of hydrogels were prepared and assembled to form a pattern for information encryption and storage (Figure 3(h)). Taking the advantages of macroscopic hydrogels, it was feasible to reassemble them to display other information. By controlling the irradiation duration under 300 nm or visible light (>450 nm), the fluorescent pattern could disappear/appear to present different information. Similarly, the reversibility of photoinduced isomerization of DAE also guaranteed that the whole information could be read out repeatedly.

### 3.3. Combination of Duration and Wavelength

Apart from duration, light with different wavelengths mentioned above can be used as the input synergistically to provide more combinations for multidimensional information encryption and storage. With orthogonality in both dimensions, the amount of the final encrypted and stored information is the product of the number of channels of wavelength and irradiation duration. In the simplest form with two wavelengths and two durations (2∗2), both of photoresponsive molecules and fluorescent molecules are required [84]. As a proof of concept, AIE molecules, 9,10-distyrylanthrance (DSA), were linked covalently with two SP moieties to obtain DSA-2SP [79]. There was a significant color change (pale yellow to blue-violet) for the reflective spectra of DSA-2SP under visible light before and after UV irradiation (Figure 3(i)). Before UV irradiation, the luminescent center was located in DSA to display yellow emission. While the excited-state energy transferred from DSA to SP to emit red fluorescence after photoinduced isomerization. Therefore, there were four kinds of color information altogether under the colorimetric and fluorometric dual readout, corresponding to four different inputs. For improving the security level of encrypted and stored information, sometimes it is essential to keep its original state invisible under natural light [85]. The dynamic fluorescent QR code composed of DAE derivatives and AIE molecules showed information invisible under two situations (under natural light before UV light irradiation and under UV light after UV light irradiation), while showing information visible under other two situations (Figure 3(j)) [80]. As a result, it is feasible to control how much information is displayed or concealed by the designed precision synthesis.

To summarize, the synthesis of photoresponsive materials is the core of multidimensional information encryption and storage in the dimension of time. It should be emphasized that the rate and conversion ratio of photoresponsive reactions have to be controlled precisely in order to obtain the changes of spectra as designed. The dynamic luminescent properties of such materials should be distinguishable before and after each irradiating interval in order to encode distinct information, which is the basis for further increasing the information capacity.

## 4. Light: Phase

The phase of light is the alternating waveform changes caused by photon vibration when light is moving forward. In order that the phase of light can be applied in multidimensional information encryption and storage, it is necessary to obtain variant phase differences at different spatial positions. Metasurfaces, composed of a layer of flat optical element (meta-atom) arrays with precise geometry, size, orientation, and arrangement at the subwavelength scale, are considered an effective structure to manipulate the phase of light in a controlled way [86–88]. Among these, the incident angle is one of the important physical quantities to control the interaction between the input light and the metasurface for phase modulation. For a designed metasurface structure, light of different incident angles indicates the different phase modulations, which can further produce multiple colors or distinct brightness to be encrypted as multiple information.

**Figure 4:**
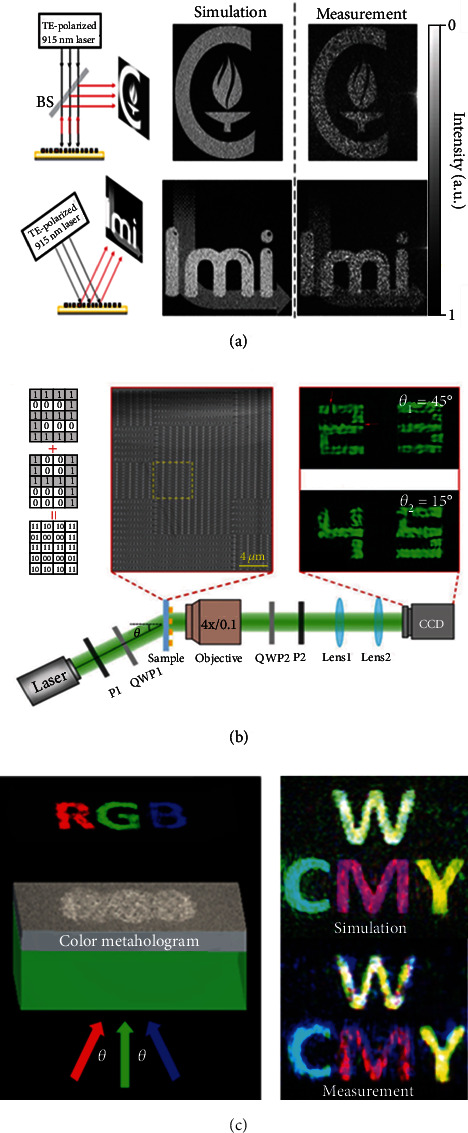
(a) Simulated and measured reflected images created by the angle-multiplexed metasurface illuminated under different incident angles (0° and 30°). (b) The design approach to encode two numbers by the structure design of coherent pixels and the optical setup for measuring the incident angle-controlled metasurface printing images. (c) Simulated and measured color holographic image produced by metasurface holograms with three primary color light at designed incident angles. Reproduced with permissions: (b) from [90], Copyright 2018, Wiley-VCH; (c) from [91], Copyright 2016, American Chemical Society.

In general, there are two kinds of metasurface images including hologram and printing images (the image printed on the structure plane). In the view of altering incident angles to project different metasurface hologram images, it is necessary to alter the case of the constant locations of the Fresnel zone boundaries. Thus, angle-multiplexed metasurfaces composed of reflective high-contrast dielectric U-shaped meta-atoms were designed for realizing the controlled independent phase response under illumination from different incident angles [89]. As shown in Figure 4(a), the hologram image of the Caltech logo displayed under the 0° illumination angle with TE-polarized light at 915 nm, while it changed to the LMI logo under the 30° illumination angle.

Unlike the metasurface hologram image, the printing image is one-to-one corresponding to the metasurface structure. It requires that each pixel of the printing images should depend on the whole elements within the pixel. So it is incapable for incident angle control when using the common multiplex pixels for the multiple printing images. Therefore, meta-atoms were designed in a novel way of coherent pixels to realize multiple printing image switching by changing incident angles [90]. The intensity of the coherent pixels was considered to be the coherent superposition contributed by all elements within it. By arranging the positions and rotation angles of the smallest element in each pixel, four types of pixels could be encoded as “00,” “01,” “10,” and “11,” respectively. After the combination of binary images of different numbers, the spatial distribution of pixel types was determined in the range of 4∗5 = 20 pixels (Figure 4(b)). At incident angles of *θ* = 45°, numbers “2” and “3” were displayed while at incident angles of *θ* = 15°, numbers “4” and “5” were shown, which demonstrated its capacity of dual-image encryption and storage.

Apart from above-mentioned single-color images produced by the metasurface, it is also feasible to obtain rich colored images under illumination at different incident angles. With both amplitude and phase modulations, full-color holographic images could be reconstructed by utilizing ultrathin plasmonic metasurface holograms made of subwavelength nanoslits [91]. Under illumination by light of three primary colors (red, green, and blue) from different incident angles, it could not only produce these three color hologram images but also generate their secondary colors (cyan, magenta, yellow, and white) depending on the combination of the input light (Figure 4(c)).

The challenge of the input light phase modulation is mainly to design metasurface structures, for producing different incident angles illumination is uncomplicated in practice. These structures should have the capacity to produce controlled phase difference according to the light with different incident angles. The method of phase modulation can also provide a higher-level information security owing to its difficulty to be counterfeited.

## 5. Light: Polarization

Polarization of light is the geometrical orientation of the oscillation of light that is perpendicular to the direction of motion. The general solution of the polarization state of light is elliptically polarized light. Under the case of the specific phase difference and amplitude ratio, it can be further classified as linear polarized light (LPL) and circular polarized light (CPL). To obtain various responses to light with different polarization states, the key is to design and fabricate desired micro/nanostructured materials with special orientation. Then, by using the correct polarization light as the input, it can display the corresponding information encrypted and stored in this polarization state.

### 5.1. LPL

**Figure 5:**
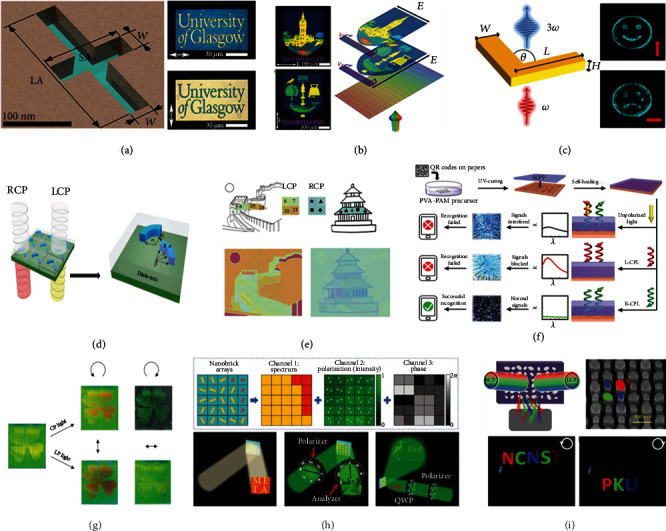
(a) A typical geometry of cross-shaped aperture and optical transmission images of the university logo with colors between the lettering and the background interconverted under the linear polarized direction of 0° or 90°. (b) Bright-field transmission images produced by the aperture array when back-illuminated with white light under LPL of the orthogonal polarization state. (c) Structure of the linearly polarizable V-shaped gold antenna for nonlinear holograms and two third-harmonic generation holographic images at 422 nm generated by vertically or horizontally polarized light at 1266 nm, respectively. (d) The transmission process of chiral color pixels under LCP and RCP illumination and the geometric structure of double-layer aluminum arcs. (e) Methods to encode two different images into one color print by designing the structure of pixels in one piece. (f) Information encryption and recognition processes of paper-printed QR codes covered by self-healing chiral photonic films. (g) Cloverleaf images composed of stratified chiral photonic polymers displayed under different polarization light. (h) Three design principles and the corresponding images of the single-celled trifunctional metasurface with triple manipulations of light. (i) Approaches of information encryption based on the metasurface with an independent channel of wavelength and polarization and its corresponding pattern presented under different input conditions. Reproduced with permissions: (a) from [92], Copyright 2016, American Chemical Society; (d, e) from [95], Copyright 2019, The Royal Society of Chemistry; (f) from [98], Copyright 2018, The Royal Society of Chemistry; (g) from [99], Copyright 2019, Wiley-VCH; (h) from [100], Copyright 2020, Wiley-VCH; (i) from [102], Copyright 2019, American Chemical Society.

The most remarkable trait of LPL is its constant polarized direction on a fixed plane. For the plasmonic cavity-aperture filter, it played a crucial part in the modulation of light by altering its geometry or composition, but it was typically composed of circular nanoholes that lacked the selectivity for LPL. Therefore, the asymmetric, cross-shaped apertures as plasmonic filters comprised of “negative” structures were designed for LPL response (Figure 5(a)) [92]. Owing to the different lengths of two arms of cross shape (long arm and short arm), each pixel showed a dual transmissive mode under the two polarization states of white incident light perpendicular to each other. The operation of these pixels was dependent on the selection rules of free-space propagating light encountering a slit. Thus, after optimizing the size and the orientation of slits and the periodicity of arrayed apertures, the desired color in the full-color range could be obtained. For the designed university logo, the long arm orientations of apertures in the lettering region and the background region were perpendicular to each other (Figure 5(a)). Under the change of the polarization state from 0° to 90°, the transmission of lettering changed the color from yellow to blue, while from blue to yellow for the background. The switchable dual-color image could effectively double the amount of information storage with different colors in a unit area.

It is noteworthy that two irrelevant full-color images encoded into a single array of cross-shaped apertures would be more practical in multidimensional information encryption and storage. In order to obtain the desired color of each nanopixel at each polarization state, the patterned white light filter containing dual polarization-dependent images was prepared by designing every pixel and their spatial distributions precisely [93]. There displayed the microimage of the University of Glasgow crest when the polarized direction was along the *y*-axis of array. After switching the direction of polarization light along the *x*-axis of array, the microimage of the university's main building appeared (Figure 5(b)). Owing to its advantages of ultrahigh spatial resolution, it is suitable for the encryption and storage of more complex information, which further protects the information from counterfeiting.

For polarization-sensitive holograms, it can be further extended to the nonlinear regime to avoid the background noise. By designing V-shaped gold antennas with different arm lengths and angles, the tunable plasmonic resonances for variable phase shifts were provided. When the nonlinear multilayer metamaterial hologram was illuminated by a linear vertically or horizontally polarized infrared laser (at 1266 nm), it generated a holographic image of a smiley or sad face at the third harmonic frequency in blue (at 422 nm), respectively (Figure 5(c)) [94].

### 5.2. CPL

The polarized plane of CPL is dynamically changing, which can be classified as left-handed circular polarized light (L-CPL) and right-handed circular polarized light (R-CPL) according to the phase difference. Similar to LPL-responsive materials because CPL can be decomposed as the combination of two LPL, it is feasible to design specific nanostructures to display different optical responses to L-CPL and R-CPL. One example of these nanostructures was the double-layer aluminum arcs embedded in a transparent polymer film as chiral color pixels (Figure 5(d)) [95]. By altering the geometries of aluminum arcs or their relative positions, each chiral color pixel could display different plasmonic colors in the whole visible region under CPL of opposite handedness, which was essentially attributed to the different plasmon coupling modes. When applying it in the multidimensional information encryption and storage, two different images to be stored were first split into different pieces according to the painting contents (Figure 5(e)). The structure designed in each piece should meet the requirement of two images simultaneously. As a result, the pattern of “the Great Wall” was only visible when the input was L-CPL, while under R-CPL illumination, the pattern of “Temple of Heaven” was visible.

Owing to the special chirality of CPL, chiral molecules are one of the promising candidates to show different responses to L-CPL and R-CPL [96]. Cellulose nanocrystals, with the ability to self-assemble into the chiral nematic structure of the left-handed helical orientation, could selectively reflect L-CPL [97]. It was used to prepare a self-healing chiral photonic film as the anticounterfeiting layer to cover the QR code on the paper (Figure 5(f)) [98]. Under natural light illumination, it displayed the indistinct QR code information on account of serious signal interference that the reflection information from two layers mixed together. When the input was pure L-CPL, the recognition of the QR code also failed because nearly the whole light was reflected on the upper layer. Only under the R-CPL illumination could it successfully reach the surface of the bottom layer to reflect the correct QR code without interference. Compared with the structures of CLP response produced by high-resolution processing methods, this chemical chirality that originate from the molecular structure is more cost-efficient and facile to prepare the CPL-responsive materials.

As mentioned above, CPL can be considered a synthetic light of two LPL or a LPL after phase shift by a quarter-wave plate. Therefore, the optimized CPL-responsive materials can present different responses to LPL simultaneously. Based on the regular chiral nematic liquid crystal materials, a distorted helical structure along the *z-*direction was engineered by stratification of an uncrosslinked liquid crystal elastomer and a chiral liquid crystal network under the linear polarized UV light [99]. The patterned area (the cloverleaf) with stratified polymer coating could reflect both CPL and LPL, but the other regions only functioned as the regular chiral nematic liquid crystals to reflect L-CPL (Figure 5(g)). Thus, only under R-CPL illumination could the surrounding area show the black background without reflection, while it displayed the green background in other cases. For the area with stratified polymer coating, there was a slight redshift of reflection wavelength under L-CPL illumination, and it kept green under R-CPL illumination. While illuminated with LPL, it mainly reflected orange light under the case of vertical LPL and green light under the case of horizontal LPL. The multiple polarization-dependent films could altogether provide five different images corresponding to different polarization light. Similarly, a metasurface with a single-cell designed structure could also show different responses to unpolarized light, LPL and CPL by spectrum, polarization, and phase manipulations of light [100]. Three different images merged into a single metasurface, acting as a structural color nanoprint, a polarization-controlled grayscale metaimage displayer, and a phase-modulated metahologram (Figure 5(h)).

### 5.3. Combination of Polarization and Wavelength

The optical materials concurrently containing the response of polarization and wavelength can be used to greatly increase the amount of information encryption and storage [40, 101]. By designing elliptically nanocylindrical meta-atoms with different shapes on the reflective-type silicon metasurfaces, six independent responsive combinations (3∗2) as the inputs were obtained, composed of two polarization light (L-CPL and R-CPL) and three wavelengths (473 nm, 532 nm, and 633 nm) [102]. On the one hand, the selective response of wavelength was dependent on the size of the elliptical surface in nanocylindrical meta-atoms, in which a larger size corresponded to the more redshifted wavelength. On the other, the selective response of polarization was determined by the major axis orientation of the elliptical surface. For demonstrating its six channels for information encryption and storage, each letter of “NCNST” and “PKU” was encoded in a special channel (Figure 5(i)). It meant that each letter only displayed under the corresponding input condition. For example, the letter “P” only appeared when illuminated by red L-CPL. Based on the combination of six independent channels, it could produce at most 63 types of different information, which obeyed the rule of 2^*N*^ − 1 combination for *N* independent channels.

Via the high-resolution processing method, it is possible to encode more refined information for polarization-responsive materials in an area of the *x*-*y* plane. Although it costs more in this way, it is more difficult to duplicate or imitate, which subsequently increases its security level of information. Polarization-responsive materials are not dependent on luminescent property, showing the great chemical stability for information storage. As long as the structures are maintained, the information hardly fades out. However, the practically available channels for different polarization states are limited. It should combine with other forms of the input light to increase the amount of information encryption and storage for higher-level information security.

## 6. Light: Others

Apart from wavelength, duration, phase, and polarization, there are still other factors to manipulate light for multidimensional information encryption and storage. In the following parts, the function of depth and power of light are discussed in sequence.

### 6.1. Depth

**Figure 6:**
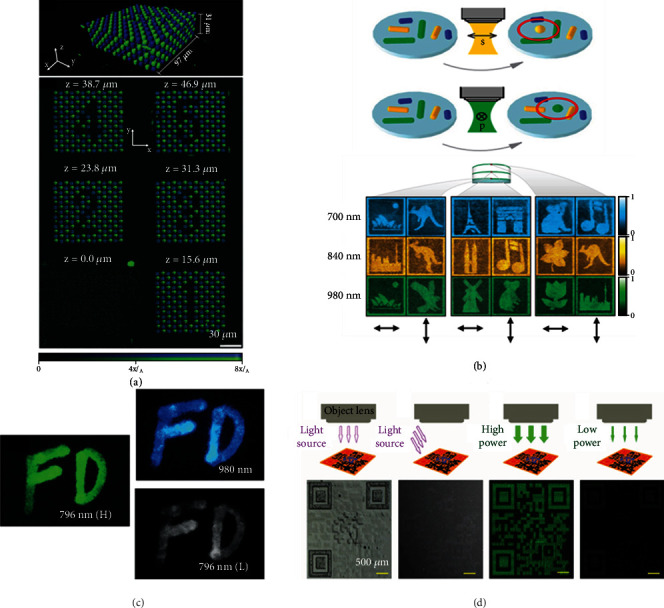
(a) Fluorescent 3D structures based on two fluorescent photoresists with blue and green emission and the individual *z-*slices of the 3D image stacked at different depths (*z* value). (b) Schematic illustration of the patterning mechanism of gold nanorods (upper) and five-dimensional optical information storage controlled by the combination of wavelength, polarization, and depth (down). (c) Three types of luminescent images of orthogonal excitation-emission UCNPs by changing wavelength or power density. (d) Four different images of the QR code displayed under four different lighting conditions dependent on the angle and the power. Reproduced with permissions: (a) from [104], Copyright 2017, Wiley-VCH; (b) from [31], Copyright 2009, Springer Nature; (c) from [29], Copyright 2016, Wiley-VCH; (d) from [30], Copyright 2016, Wiley-VCH.

Depth here indicates that the incident light can be focused on the *z-*plane with different depths to show the information of each layer in a 3D structure. To summarize the examples mentioned in previous sections, it is not difficult to find that most of the examples for multidimensional information encryption and storage are operated in a 2D structure in the *x-y* plane, whether it is a film, hydrogel, or metasurface. The importance of another spatial dimension—depth (*z-*axis)—is not represented in these works. Compared with common 2D structures, it is obvious that 3D structures of information encryption and storage provide a higher security level against counterfeiting [103]. Under the devices with high resolution in the *z-*axis, it is easy to control light to illuminate different planes along the *z-*axis to display information at different depths. In order to evaluate this method, 3D structures with different information along the *z-*axis were prepared by 3D laser lithography [104]. The nonfluorescent 3D cross-grid with nominal lattice periods and fixed layer spacing was selected as the 3D-structured substrate. CdSSe/ZnS core-shell quantum dots, as the fluorescence markers, were mixed with a photoresist to be added step by step into the 3D cross-grid to form different figure information along the *z-*axis. Measured by confocal laser scanning fluorescence microscopy, figures from “1” to “5” sequentially displayed clearly along the *z-*axis from the bottom to the top (Figure 6(a)).

Furthermore, considering the depth as one of the input dimensions, a five-dimensional optical system for information encryption and storage, with the dimensions of wavelength, polarization, and three-dimensional space, was achieved [31]. To obtain orthogonality in all dimensions, the gold nanorods with the properties of longitudinal surface plasmon resonance, represented by an excellent wavelength and polarization sensitivity, were required. During the process of photothermal patterning, different linear polarized laser pulses could solely be absorbed by specific gold nanorods that met two requirements (Figure 6(b)). One was that the long-axis orientation of gold nanorods should be identical with the polarized direction of a laser pulse, and the other was that the absorption cross section of gold nanorods should match the wavelength of laser pulse. The amount of information encryption and storage was dependent on the number of stacking layers, the sort of gold nanorods with different long-axis orientations, and the absorption cross sections on each layer. With the three recording layers separated by two transparent spacers, it could totally exhibit 18 patterns (3∗2∗3), with three channels in wavelength (700 nm, 840 nm, and 980 nm), two channels in polarization (horizontal and vertical polarization), and three channels in depth. Therefore, through adding the depth information in the spatial dimension, the performance of information encryption and storage is highly improved, which is extremely beneficial for high-density optical information storage.

For multiple information stacked along the *z-*axis in one material, it usually needs the optical collecting benchtop instruments with good *z-*axis resolution to read out each layer of information for protection from the mutual interference. The development of portable optical collecting systems with better *z-*axis resolution will effectively expand the practicability of the method for multidimensional information encryption and storage in different depths. In essence, the most important issue is to make sure that each layer information along the *z-*axis can only be observed under one specified input light. Any novel materials or strategies meeting this requirement are potentially used to fabricate 3D-structured information.

### 6.2. Power

It is well known that for luminescent materials, the emission intensity is related to the power of incident light. By means of selectively controlling the luminescence mode under light irradiation with various power values, different information can be read out. For designed UCNPs with core-multishell structure (NaGdF_4_:Yb,Er@NaYF_4_:Yb@ NaGdF_4_:Yb,Nd@NaYF_4_@NaGdF_4_:Yb,Tm@NaYF_4_), it could concurrently show two types of emissions with the NIR DCL and green UCL under the high-power density light of 796 nm (>0.5 W/cm^2^) (Figure 6(c)) [29]. By using the low-power density light with the same wavelength (<0.5 W/cm^2^), however, the part of UCL almost disappeared, with only DCL remaining. Meanwhile, it had significant orthogonal excitation-emission properties, showing green emission at 796 nm excitation and blue emission at 980 nm excitation, respectively. Wavelength and power of light could be jointly considered as the inputs to generate a pattern containing three different information.

Besides, it could also be achieved by the integration of multiple materials with different light-power sensitivities. When photonic crystal dots containing rhodamine 6G were controllably shaped as bumps, plates, and coffee rings on the substrate, they showed different fluorescence enhancement abilities [30]. After printing three kinds of photonic crystal dots to assemble a QR code, under high power excitation, the whole photonic crystal dots emitted strongly and the image of the complete QR code appeared (Figure 6(d)). When decreasing power to 60%, only bump-shaped photonic crystal dots (finding the pattern area on the QR code) could be observed because they had the strongest fluorescent enhancement ability. These photonic crystal dots also reacted differently to light from the top and from the side, so the incident angle could be considered another dimension of the input light. By the combination of incident angles and power of light, the anticounterfeiting QR code with quadruple images was achieved.

Although it is maneuverable to adjust the power of light, there still exist challenges such as severe local heating when using high-power light. The instability of luminescent materials under high-power light irradiation will affect the successful information readout, which limits its application to some extent.

## 7. Summary and Perspective

In conclusion, multidimensional information encryption and storage when the input is light have been proven as an effective and reliable approach to increase the security level of information. Six-dimensional features of the input light (wavelength, duration, phase, polarization, depth, and power) could be used separately or synergistically to encrypt and store multidimensional information in one material. It is simple and practical to modulate light to meet the requirements of different inputs. The pros and cons of these six-dimensional features are summarized in Table 1. Based on the features of different input light, we can purposefully design and prepare suitable materials with special structures. These materials have the capacity to output the predesigned information under the corresponding input light. The realization of multidimensional information encryption and storage in one material can effectively improve the security level of information, showing a broad market perspective.

Though decent progress has been achieved, it is still essential for us to think deeply about the current challenges. First, it is still not achievable to take full advantage of multidimensional characteristics of light for safer information encryption and storage. On the basis of information encrypted and stored in one-dimensional feature of light, other dimensional features of the input light can be synergistically introduced to realize the multidimensional information system. Thus, the crosstalk problem among different information should be placed much emphasis, which requires great resolving ability to distinguish the small enough discrepancy to decrypt as different information. In other words, the preparation of materials or the design of nanostructures with multiple functions should be more precise and more controllable. Second, the stability of information storage or readout is rather important, which has a direct impact on the quality of information output. The irreversible photobleaching phenomenon may appear when luminescent materials are exposed under UV light with high power for a long time. It should have the capacity not only to keep for a long time under the ambient condition but also to obtain stable information readout under the special input conditions. Third, the available and portable way for information readout is the key factor for expanding its application in daily life, even though it may be a sophisticated structure or the extremely special material. It is important to keep high-level information security and good practicability simultaneously. Other technologies for simplifying or miniaturizing the device for the input or the output will continuously drive this field to make tremendous progress. Last but not least, different from the research on the lab, the economic cost is more worthy of consideration for every manufacturer in practical application. Because the eventual goal of information security is to gain more benefits, there will be a comparison between the cost of different information security technologies and the profits of information itself. Cost-efficient preparation methods of novel materials suitable for multidimensional information encryption and storage are strongly required.

Therefore, more efforts on the multidimensional information encryption and storage with light as the input are needed. To summarize, future innovative works should continue to aim at the acquisition of high-level information security through three aspects: the input, the material, and the output. For the input side, as mentioned in Introduction, light is not the sole choice. Other forms of physical signals such as force, heat, electricity, magnetism, or chemical signals could be used together as the multiplexed input. A variety of inputs indicate more channels for different kinds of information encryption and storage to provide high-level information security. Then, the selected input is closely related to the material. Researches about novel functional/smart material will provide greater possibility to find the optimal one to meet the requirement of the input. By introducing fabrication methods with high spatial resolution, it is possible to make the size (shape) of the smallest information unit further downsize (refine). It is an important basis to realize high-density information storage and assure information readout with high quality. For the output side, information readout should be more efficient and more completed. For example, it can be considered not only as colored pattern information but also as the information of spectral pattern that contains more channels for information encryption and storage. Therefore, the rapid development of integrated process technologies, preparation of multifunctional materials, and information sensing technology will dramatically promote the capacity to control the input, the interaction between the input and materials, and the output. With the ingenious encryption to store multidimensional information in one material, these methods for information encryption and storage will play an important role in the future of information security.

## Figures and Tables

**Table 1:** Pros and cons of six-dimensional features of the input light for multidimensional information encryption and storage.

	Pros	Cons
Wavelength	(1) Easy to prepare (2) Suitable for pattern printing	(1) Photoluminescence stability (2) Background interference (3) Light source (4) Mixing problem

Duration	(1) Dynamic (2) Information only for single-time read (3) Suitable for pattern printing	(1) Information relevance (2) Longer readout time (3) Adaptable

Phase	(1) Difficult to counterfeit (2) Easy to read (3) High-level information security	(1) Complex design (2) Nanofabrication (3) Cost

Polarization	(1) Difficult to counterfeit (2) High-level information security	(1) Complex design (2) Nanofabrication (3) Cost (4) Requires polarizer (quarter-wave plate)

Depth	High-density information storage	(1) Requires a device with good *z*-axis resolution (2) Low efficiency for information readout

Power	(1) Easy to modulate (2) Suitable for pattern printing	High power to induce information damage

## Data Availability

No data were used to support this study.
